# Adipocyte Apoptosis Following a Novel Method for Double Chin Reduction: A Pilot Human Histology Study

**DOI:** 10.1111/jocd.16643

**Published:** 2024-11-13

**Authors:** David J. Goldberg

**Affiliations:** ^1^ Division of Schweiger Dermatology New York NY USA

**Keywords:** apoptosis, double chin, fat reduction, submentum

## Abstract

**Background:**

Submental fullness is perceived as unattractive by both men and women. The noninvasive simultaneous delivery of HIFES and synchronized radiofrequency+ (Sync RF+) technologies aims to address the submental fullness by concurrently targeting the skin, adipose tissue, and weakened anterior belly of the digastric muscle, the three contributing layers to the double chin appearance.

**Aims:**

This study aims to investigate the histological changes to adipose tissue related to cell morphology, caspase‐7, and Bcl‐2 levels to detect adipocyte apoptosis following the HIFES and Sync RF+ treatment on human subjects.

**Methods:**

The active group (*n* = 6) received single 20‐min treatment on the submental area, while the control group (*n* = 2) did not receive any treatment. Biopsies of subcutaneous fat tissue were obtained at baseline, and 24 h and 7 days posttreatment. The specimens were histologically and immunohistochemically analyzed for changes in morphology, caspase‐7, and Bcl‐2 levels.

**Results:**

Observed caspase‐7 levels increased by 511% at 24‐h posttreatment, and 101% at 7 days (*p* < 0.0001), while the Bcl‐2 levels decreased by 89% at 24 h and 24% at 7 days posttreatment (*p* < 0.0001). The control group had no statistically significant relative changes in the activity of caspase‐7. Posttreatment adipocytes were shrunken in size, and shapes lost their uniformity compared to baseline. Five of six subjects reported the treatment as being comfortable. No adverse events were observed during the study.

**Conclusions:**

The results of this human histology study indicate that noninvasive HIFES and Sync RF+ technologies have a favorable safety profile for submental fat reduction through the induction of adipocyte apoptosis.

**Trial Registration:**

ClinicalTrials.gov identifier: NCT06282172

## 1 Introduction

Submental fullness, commonly known as a “double chin,” is widely regarded as unappealing by both men and women [1]. It is an indication of higher body fat content or aging, traits typically deemed undesirable in today's society [2]. In a culture that values youthfulness and promotes a slender physique, the presence of a double chin can be a source of dissatisfaction for many individuals.

The appearance of a double chin is affected by the skin, fat tissue, and weakened anterior belly of digastric muscle, all of which are altered by the aging process [3, 4]. Skin with age undergoes dermal atrophy with a gradual loss of glycosaminoglycans, collagen, elastic fiber, and their structural disorganization, which externally manifests as skin laxity [5]. The muscle tone of the digastric muscle, the submental muscle layer representative, decreases over time [6, 7], resulting in laxity and protrusion to the submental area. On the other hand, fat tissue accumulates extensively due to age‐related fat hypertrophy and gravitational repositioning caused by weakened supporting tissue and underlying facial ligaments [8]. While weight gain plays a role in fat tissue hyperplasia in the submental area, aging and genetically predisposed fat distribution oftentimes do not correlate with the total body fat content [9]. Consequently, relying solely on weight loss to reduce submental fullness may be ineffective [10]. This creates a need for solutions specifically targeting submental fullness.

Both surgical and nonsurgical options are currently available for the reduction of submental fullness. While invasive approaches have proven efficacy, they carry the drawbacks of surgical recovery time and the potential for side effects and adverse events. Reported side effects of invasive procedures include pain and discomfort, infection, nerve injury, scarring, swelling, and hematoma [11]. To minimize treatment‐related downtime and adverse events, nonsurgical methods have been developed. Although side effects and adverse events are reduced with nonsurgical technologies, they are not without risks. Described side effects of deoxycholic injection are nerve injury, dysphagia, ischemia and skin necrosis, or bruising [12]. Cryolipolysis, on the other hand, carries the risk of rare but concerning paradoxical adipose hyperplasia [13].

Generally, all facial muscles, including the digastric muscle, have been dismissed in nonsurgical methods. Recently, a noninvasive technology combining HIFES for facial muscle stimulation with synchronized radiofrequency (Sync RF) for simultaneous skin rejuvenation has been introduced to treat the forehead and midface. The technology has been shown to be effective in reducing wrinkles, improving skin evenness, and providing a face‐lifting effect [14, 15]. Currently, a new application of this combined technology focused solely on the submental area has been introduced. It uses HIFES to target the lax digastric muscle and Sync RF+ with stronger power than in facial treatments to reach higher temperatures in the fat layer for its reduction along with skin rejuvenation.

Radiofrequency (RF) has been a documented safe method for various indications, notably for skin rejuvenation and fat reduction. Radiofrequency selectively increases the temperature in targeted tissue based on their impedance differences [16]. The heat‐induced collagen coagulation, reorganization of collagen and elastic fibers, and their renewed formation contribute to skin rejuvenation [17]. Selective heating of fat tissue induces adipocytes to undergo apoptosis through hyperthermic stress [18], an inflammatory‐free fat reduction pathway. Meanwhile, HIFES technology addresses the delicate digastric muscle using the electric field, triggering brain‐independent supramaximal contractions when delivered to the mylohyoid nerve, the innervation of the anterior belly of the digastric muscle. The muscle load activates dormant satellite cells and heat shock proteins [19, 20], essential elements for reversing muscle atrophy, ultimately restoring muscle fiber thickness and tone.

This pilot study aims to investigate the submental fat reduction aspect of combined HIFES and Sync RF+ energies on the submental area by observing histological changes to adipose tissue related to cell morphology, as well as caspase‐7 and B‐cell lymphoma 2 (Bcl‐2) levels for adipocyte apoptosis detection. Moreover, safety of the procedure and subject's treatment comfort were explored.

## 2 Materials and Methods

This prospective, single‐center, two‐arm, open‐label pilot study enrolled 8 subjects, who were randomly allocated into active and control groups in a ratio of 6:2 (active:control) using the Microsoft Excel software (Microsoft, Redmond, WA). The inclusion criteria were adult subjects seeking treatment of the submental fullness and willingness to undergo a biopsy in the submental area. Among the exclusion criteria were any condition that contraindicated the application of radiofrequency and electrical field in the submental area, that is, local infection in the treated area, skin‐related autoimmune diseases, metal implants, acute neuralgia or neuropathy, pregnancy or nursing, and kidney or liver failure. Subjects were educated about the study treatment and procedures and potential risks and benefits, and signed a written informed consent. The study was conducted from May 2023 till December 2023.

The active group received one treatment with intensities of HIFES and Sync RF+ energies set to a maximally tolerated level according to the subject's feedback. The 20‐min treatment was performed using the EMFACE device (BTL Industries Inc., Boston, MA) with a self‐adhesive novel submentum applicator. After the treatment, a 5‐point Likert scale (1 = strongly disagree, 5 = strongly agree) Therapy Comfort Questionnaire, which includes a 10‐point numerical analog scale for pain (NAS, 0 = no pain, 10 = worst possible pain), was given to the treated subjects for therapy comfort evaluation. The control group was treated with a sham device but did not receive any actual treatment. Safety of the procedure was assessed by the observation of any adverse events or side effects during the course of the study.

The follow‐up (FU) visits were scheduled at 24 h, 7 days, and 14 days posttreatment. The primary outcome measures included histological evaluation of the subcutaneous fat tissue from the obtained biopsy specimens. The punch biopsies (3 in diameter and 10 mm in depth) of the skin, including subcutaneous fat tissue from the submental area, were obtained at the baseline visit, the 24‐h FU, and the 7‐day FU. The timeline of the control group's biopsies was scheduled accordingly to mimic the biopsy timing of the active group. Before the biopsy, a topical anesthetic (1% lidocaine) was applied to the subjects to reduce potential discomfort. After the biopsy, the wounds were closed and disinfected, and subjects were given instructions about wound after‐care. The 14‐day FU was scheduled as a safety precaution to ensure biopsy wounds were properly healed.

The obtained biopsy specimens were then fixed in 10% neutral buffered formalin (NPB), embedded in paraffin wax, stained, and sliced by a microtome to 5 μm thickness. The specimens were stained using the hematoxylin–eosin (H&E) method for the histological morphology evaluation. The immunohistochemical (IHC) method was performed for the detection of proapoptotic marker caspase‐7 protein and antiapoptotic marker Bcl‐2 protein using the following antibodies: monoclonal rabbit anti–caspase‐7 antibody (Merck, Rahway, NJ, USA) and monoclonal mouse anti‐hu Bcl‐2 antibody (Exbio, Prague, Czech Republic). Initially, slides were transferred into a container with a citrate buffer for antigen retrieval. The container was then placed in a pressure cooker filled with distilled water, heated to 95°C for 15 mins, and later cooled down. Once cooled, the slides were washed in phosphate–buffered saline (PBS) buffer and blocked with either 2% BSA to prevent nonspecific binding. Next, primary antibodies are applied and left to incubate overnight at 4°C. After washing the slides with PBS, secondary antibodies diluted in PBS (1:500) with a fluorophore were added for 1 h at room temperature. Following another round of PBS washing, the slides were stained with DAPI (1:500) for 10 min in the dark at room temperature. They were then washed in PBS, rinsed in distilled water, and mounted with an aqueous mounting medium. Finally, coverslips were secured using nail polish. IHC staining resulted in fluorescence captured and photographed by an automated fluorescence microscope (Olympus IX83; Olympus, Tokyo, Japan).

To address the issue of limited sample size, three histological slices were examined from each subject for every evaluation method used. A blinded clinical histopathologist evaluated the H&E‐stained slices for cell morphology, as well as the IHC‐stained slices for the presence of caspase‐7 and Bcl‐2 in the ROI 327 648 × 20 478 μm without the knowledge of which group the specimens belonged to or which study visit was the sample from. The IHC staining resulted in fluorescence of all the nuclei in the color blue (DAPI) and positively stained caspase‐7 and Bcl‐2 proteins in green fluorescence. The presence of caspase‐7 or Bcl‐2 was normalized as relative fluorescence to total DAPI fluorescence. This value was then used for the relative change calculation to determine the shift of caspase‐7 and Bcl‐2 quantity in the adipose tissue.

A two‐way ANOVA test (GraphPad Prism; GraphPad Software, Boston, MA) was performed for the statistical analysis when comparing posttreatment data to baseline. The level of statistical significance was considered as *α* = 0.05.

## 3 Results

Eight subjects (*n* = 8) were enrolled in this study. Six subjects (*n* = 6, females, age 51.8 ± 11 years, BMI 26.1 ± 2.4 kg/m^2^, and Skin Type II–V) were allocated to the active group and underwent one study treatment on the submental area. The average HIFES energy applied was 65% ± 28.11% of its intensity, while the RF intensity was set to 100%. Two subjects (*n* = 2, females, 56 and 60 years, BMI 22.7 and 27.44 kg/m^2^, and Skin Type II and IV) served as control and did not receive any treatment. All subjects completed the study's scheduled visits, and there were no withdrawals from the study. No adverse events or side effects were observed during the course of the study. The biopsy wounds of both groups healed without any complications.

Histological evaluation of H&E‐stained slides revealed morphological changes in the posttreatment fat tissue. Adipocytes were shrunken in size and shape and lost their uniformity compared to baseline adipocytes, which were uniform in size and polygonal shape with intact septae.

**FIGURE 1:**
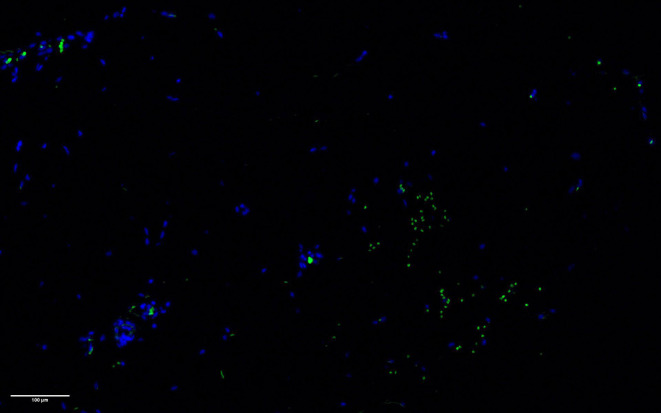
Staining for caspase‐7 resulted in increased fluorescence (in green color) at 24‐h FU (below) posttreatment compared to baseline (above). Blue fluorescence (DAPI) represents adipocyte nuclei, while green fluorescence represents positively stained caspase‐7 protein.

**FIGURE 2:**
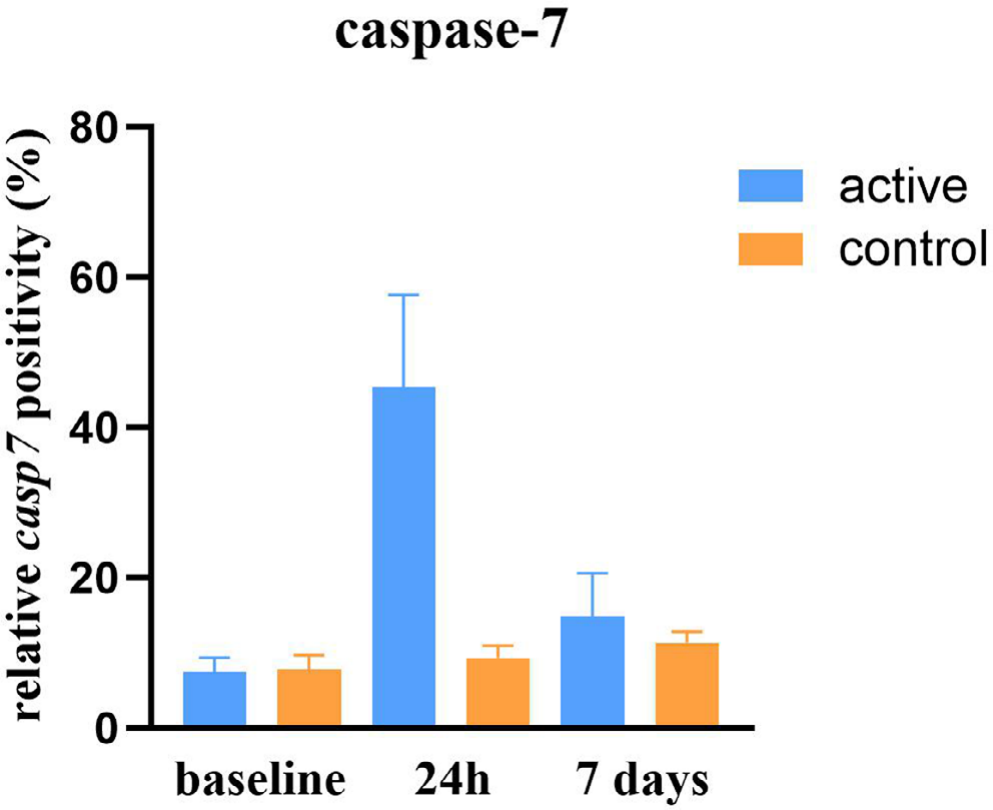
Caspase‐7 positivity relative to total DAPI positivity (%) of active (right) and control (left) group.

**FIGURE 3:**
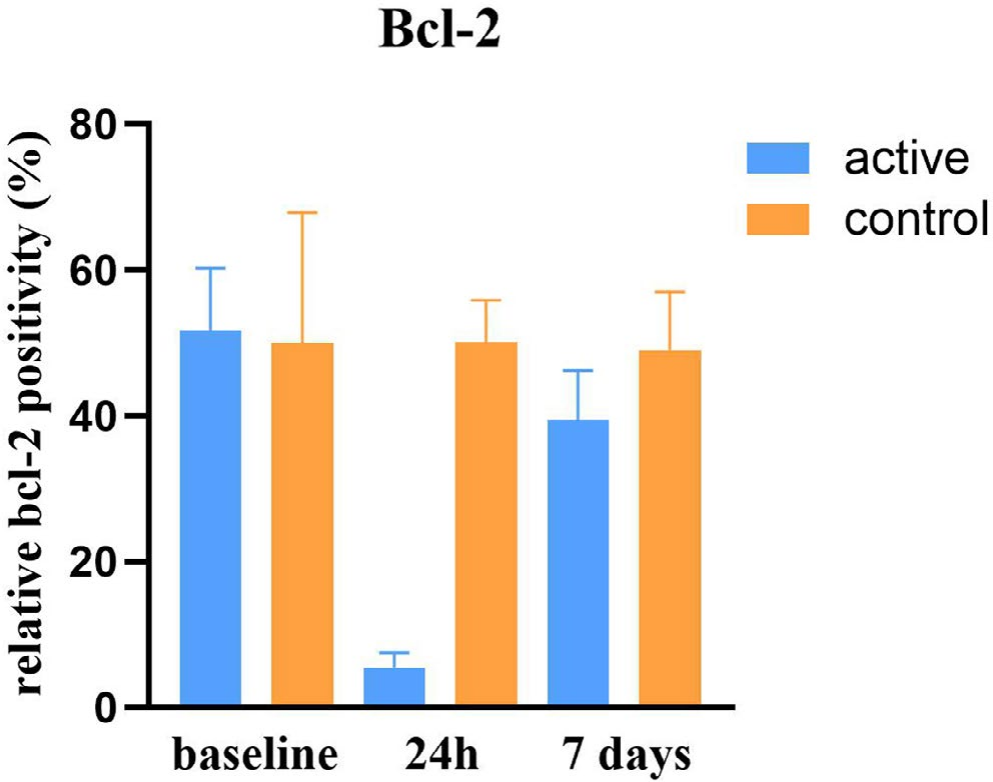
Bcl‐2 positivity relative to total DAPI positivity (%) of active (right) and control (left) group.

Observed caspase‐7 positivity increased (*p* < 0.0001) after treatment, peaking at the 24‐h FU with a relative change of +511%. At the 7‐day FU, levels decreased to +101% compared to baseline (see Figures 1 and 2). While caspase‐7 levels were elevated, Bcl‐2 levels were downregulated, showing a relative change of −89% at the 24‐h FU and −24% at the 7‐day FU posttreatment (*p* < 0.0001; see Figure 3). The control group had no statistically significant changes in caspase‐7 positivity (*p* = 0.128, relative change of +21% at 24 h and +45% at 7 days) or Bcl‐2 levels (*p* = 0.985, relative change of 0% at 24 h and −2% at 7 days).

Five of six treated subjects considered the treatment comfortable, and most (83%) reported perceived discomfort ranging from no pain at all to mild pain (on average 1.5/10 points on NAS).

## 4 Discussion

This study investigated the change in the levels of caspase‐7, Bcl‐2 apoptosis markers, and the adipocyte morphology following the treatment delivering simultaneously HIFES and Sync RF+ technologies on the submental subcutaneous adipose tissue. The changes were pronounced mostly at 24 h posttreatment for all studied parameters. Caspase‐7 levels increased at 24 h FU by 511% and by 7 days by 101%, while the Bcl‐2 levels decreased by 89% at 24 h and 24% at 7 days. Adipocyte morphology was affected by the study treatment as well, showing a reduction in size and loss of shape uniformity. The treatment was perceived as comfortable by the majority of participants. No adverse events were detected during the course of the study.

Posttreatment induction of adipocyte apoptosis is suggested by the heightened presence of caspase‐7 and reduced Bcl‐2 levels. Bcl‐2 is an antiapoptotic molecule, sustaining cells alive by inhibiting cytochrome‐c release and the subsequent caspase activation [21]. Increased Bcl‐2 protein has been previously reported to contribute to apoptosis resistance of mature adipocytes [22]. A study by Nagel et al. [22] investigated whether downregulation of Bcl‐2 would on that account sensitize adipocytes to apoptosis. The 85% downregulation of Bcl‐2 has been demonstrated to lead to a 25% higher sensitivity to apoptosis. The decreased levels of 89% observed in this study are therefore suggested to improve adipocyte susceptibility to apoptosis, which is, on the other hand, executed by the caspase‐7 protein. Caspase‐7 is an enzyme responsible for the cleavage of crucial proteins necessary for maintaining cell integrity. The cleavage results in nuclear condensation, genomic DNA fragmentation, and the formation of apoptotic bodies for the final cell degradation by macrophages [23]. The combined HIFES and Sync RF+ effect on adipose tissue was furthermore observed in the morphological change of adipocytes. The shrunken size implies fat reduction following the treatment. With no observed signs of necrosis, the statistically significant alteration of apoptotic markers, and histological findings, the study results indicate ongoing apoptosis in the submental fat tissue following the HIFES + Sync RF+ treatment.

Radiofrequency has previously been suggested to induce adipocyte apoptosis. This present study examined the specific effects on submental fat tissue following the simultaneous delivery of HIFES and Sync RF+ technologies. Apoptosis can be considered the desired fat reduction pathway for its irreversibility and safety. Unlike necrosis or inflammation, apoptosis is a highly controlled cell death process and does not negatively affect the surrounding healthy tissue [23], which can cause side effects such as swelling, bruising, and pain described in submental fat reduction using deoxycholic acid or cryolipolysis [24]. Previous studies have demonstrated that inflamed adipocytes in the fat tissue observed in obese patients have a negative impact on overall health, taking part in insulin resistance and even instigating inflammation in remote organs [25]. Fat reduction by the induction of apoptosis, therefore, limits the inflammatory response [23], avoiding negative health consequences while providing long‐term results through the death of adipocytes.

Due to the invasive nature of biopsies, the sample size was limited to eight subjects. However, the sample size was sufficient to demonstrate the hypothesized trend in the treated tissue with statistical significance. For a more comprehensive understanding, a larger sample size, including a wider range of BMI and age, and longer FUs are recommended.

Despite the limitations, the key strength of this study was the observance of the changes on the molecular level, including both caspase‐7 and Bcl‐2 proteins for investigating apoptosis occurrence, as well as on the histological level via cell morphology examination. Prior evaluations have shown the associated histologic changes.

Additionally, besides adipose tissue, the combined HIFES and Sync RF+ energies have the potential for submental contouring by targeting the skin and the anterior belly of the digastric muscle. Therefore, future studies are needed to assess the effects of this technology on these tissues.

## 5 Conclusions

The results of this investigation implied apoptosis as the mechanism of lipolysis following the noninvasive simultaneous delivery of HIFES and Sync RF+. The treatment was safe and perceived as comfortable by the study participants.

## Author Contributions

Author D.J.Goldberg carried out the experiment, analyzed results, and wrote the manuscript.

## Ethics Statement

The author confirm that the ethical policies of the journal, as noted on the journal's author guidelines page, have been adhered to. The study was approved by the Advarra Institutional Review Board on May 24, 2023. All subjects voluntarily participated and signed a written informed consent.

## Conflicts of Interest

The author declares no conflicts of interest.

## Data Availability

The data that support the findings of this study are available on request from the corresponding author. The data are not publicly available due to privacy or ethical restrictions.
